# Integration of Molecular Data in the Prognostic Stratification and Management of Endometrial Carcinoma

**DOI:** 10.3390/jpm12122083

**Published:** 2022-12-18

**Authors:** Antonio Raffone, Antonio Travaglino, Diego Raimondo, Paolo Casadio, Renato Seracchioli, Gian Franco Zannoni, Antonio Mollo

**Affiliations:** 1 Division of Gynecology and Human Reproduction Physiopathology, IRCCS Azienda Ospedaliero-Universitaria di Bologna, 40126 Bologna, Italy; diego.raimondo2@unibo.it (D.R.); paolo.casadio@unibo.it (P.C.); renato.seracchioli@unibo.it (R.S.); 2 Department of Medical and Surgical Sciences (DIMEC), University of Bologna, 40126 Bologna, Italy; 3 Pathology Unit, Department of Woman and Child’s Health and Public Health Sciences, Fondazione Policlinico Universitario Agostino Gemelli IRCCS, 00168 Rome, Italy; antonio.travaglino@guest.policlinicogemelli.it (A.T.); gianfranco.zannoni@unicatt.it (G.F.Z.); 4 Pathology Unit, Department of Advanced Biomedical Sciences, School of Medicine, University of Naples Federico II, 80138 Naples, Italy; 5 Pathology Institute, Catholic University of Sacred Heart, 20123 Rome, Italy; 6 Gynecology and Obstetrics Unit, Department of Medicine, Surgery and Dentistry “Schola Medica Salernitana”, University of Salerno, 84084 Baronissi, Italy; amollo@unisa.it

**Keywords:** endometrial carcinoma, The Cancer Genome Atlas, genomics, biomarkers, guidelines, MMR, NSMP, TP53, copy number, mutational load

## Abstract

In the last years, the TCGA-based molecular classifier have been progressively integrated in the management of endometrial carcinoma. While molecular assays are increasingly available across pathology laboratories, the additional costs will expectedly be compensated by a reduction in overtreatments and a prevention of recurrences. The additional time might be shortened by assessing molecular markers on biopsy specimens. Retrospective data suggest that the molecular classifier will have a major impact of on the risk stratification, with many patients having their risk class down- or upstaged based on *POLE* mutations or p53 abnormalities, respectively. However, there are still several issues to be resolved, such as the prognostic value of the TCGA classifier in each FIGO stage, or the type of adjuvant treatment most suitable for each molecular group. Other issues regard the prognostic stratification of the mismatch repair-deficient and “no specific molecular profile” groups, which currently follows the same criteria; however, the former seems to be prognostically consistent regardless of FIGO grade and histotype, whereas the latter appears highly heterogeneous. Numerous clinical, histological, immunohistochemical and molecular markers have been proposed to refine the TCGA classification, but their prognostic value is still undefined. Hopefully, prospective data collected in the next years will help resolving these issues.

The last years have seen an unprecedented revolution in the approach to endometrial carcinoma (EC). Such revolution began with the publication of the study by The Cancer Genome Atlas (TCGA) in 2013, which showed that EC could be subdivided into 4 molecular prognostic groups based on mutational burden and somatic copy number variations. Since then, numerous studies have contributed to the translation of the TCGA findings into routinary usable protocols for the management of EC [1,2,3,4].

A crucial step in this process has been the validation of cheaper and easier surrogates of the complex and expensive molecular analyses performed by TCGA. Therefore, the use of immunohistochemistry for mismatch repair (MMR) proteins and p53 protein and of polymerase-ε sequencing has been found able to replace the molecular assessment of mutational burden and copy number variations. On this account, EC can now be subdivided into the “MMR-deficient” group (surrogate of the “hypermutated” group), the “*POLE*-mutant” group (surrogate of the “ultramutated” group), the “p53-abnormal” group (surrogate of the “copy number-high” group), and the “no specific molecular profile group” (NSMP, surrogate of the “copy number-low” group). These surrogates have allowed several research groups to test the TCGA classification on their EC series and confirm its prognostic value [2,3,4,5].

Given that the TCGA study only included endometrioid and serous EC, several studies have assessed the molecular classification on other histotypes, including clear cell, carcinosarcoma, and undifferentiated/dedifferentiated carcinoma, consistently showing a good prognosis for *POLE*-mutant cases and poor prognosis for the p53-abnormal cases [2,5]. The TCGA classification has also been tested in different ESMO risk categories as defined by clinicopathological variables [4,6]. Numerous additional clinical, histological, immunohistochemical, and molecular markers have been proposed to substratify the TCGA groups in general or in specific subsets of EC [4,7,8,9,10,11,12].

In 2020 (7 years after the publication of the TCGA study), the updated ESGO-ESTRO-ESP guidelines adopted the molecular prognostic classification in the risk stratification of EC. Two crucial updates regard (i) *POLE*-mutant ECs, which are categorized as “low-risk” when confined to the uterus, regardless of any other clinicopathological or molecular factors, and (ii) p53-abnormal ECs, which are categorized as “high-risk” in the presence of myoinvasion, regardless of any other factor [2,13].

As observed by Oberndorfer et al. in their recently published paper [14], a high percentage of patients (more than 50% in their series) have their risk class up- or downstaged with the molecular classification compared to the ESMO classification. This is in agreement with previously published results based on retrospective data [15] and indicates a major impact of the TCGA classification on the management of EC.

Possible limiting factors of the TCGA classifier discussed in the Literature are the additional costs and the limited availability of molecular analyses. In this regard, limiting *POLE* analysis to a subset of patients [16], or even not assessing *POLE* at all [17], have been studies as possible (although suboptimal) solutions. However, it should be noted that molecular analyses are becoming more and more widely available across pathology laboratories, and that oncologic patients are usually referred to highly equipped specialized centers. Moreover, as highlighted by Oberndorfer et al. [14], the additional costs will expectedly be compensated by a reduction in unnecessary treatments on the one hand and a reduction in recurrences on the other hand. Data regarding the cost-effectiveness of the TCGA classification will be available in the next few years.

Another issue raised by Oberndorfer et al. is the additional time required for molecular analyses (18.5 days on average in their series) [14]. This leaves oncologists with a tight timeline to define the optimal adjuvant treatment. A possible solution to this problem could be to perform the analysis of *POLE* on diagnostic biopsy specimens. In fact, it has been shown that there is high concordance in the assignment of molecular groups between biopsy and hysterectomy specimens [18,19]. In order not the delay the first diagnosis and hysterectomy, the pathological report of the biopsy specimen should be provided without waiting for the results of *POLE* analysis. Regarding immunohistochemistry, it appears advisable to perform it on the hysterectomy specimen, as it might reveal, for example, a subclonal p53-abnormal component [5].

Apart from the problems highlighted by Oberndorfer et al., there are further issues that have been raised with the use of the molecular classifier. Amant et al. highlighted that it is still unclear how the TCGA classifier impacts the prognosis in each FIGO stage, and that prospective studies are needed to resolve this point. Furthermore, they underlined that the type of adjuvant treatment most suitable for each group is not defined [20].

Merlotti et al. raised the question whether it is reasonable to treat p53-abnormal low-grade ECs with superficial myoinvasion as high-risk tumors [21]. These cases are considered to be exceptionally rare [22], but they have been described in the literature. Hachishuga et al. suggested that p53-abnormal low-grade ECs may occur in elderly patients and may be associated with worsened prognosis [23]. According to Vermij et al., the TCGA classification is unlikely to change the prognosis in ESMO low-risk ECs [24]. Clearly, it is necessary to collect series of these cases to define their frequency and their biological behavior.

Another issue regards whether it is meaningful to substratify MMR-deficient ECs according to FIGO grade and histotype [2,5]. In fact, there are data suggesting that there are no such differences, and that only SWI/SNF-deficient undifferentiated/dedifferentiated ECs show highly aggressive behavior regardless of MMR deficiency [7,25,26,27,28]. In addition, it seems that MMR-deficient ECs with MLH1 promoter methylation are more aggressive than ECs with mutations in the MMR genes [27], but it is unclear if this may require a different treatment.

The prognostic heterogeneity of NSMP ECs appears as another unresolved issue. Oberndorfer et al. adopted the classification proposed by Stelloo et al. for ESMO high-intermediate risk ECs, which involves CTNNB1 exon 3 mutation and L1CAM overexpression [4,14]; these markers are currently being tested in the PORTEC-4 trial, but are currently not included in the ESGO-ESTRO-ESP guidelines [29]. Other prognostic stratifications of NSMP ECs have been proposed for other ESMO risk groups or regardless of the risk group [7,10,11]; however, their clinical value is still unclear. The scenario is further complicated by the plethora of additional clinicopathological, immunohistochemical and molecular markers that have been proposed to refine the TCGA groups [8,9,12,30].

Hopefully, the prospective and large-scale assessment of the TCGA classification will allow resolving these issues and obtaining the necessary data for an optimal management of EC.

## Data Availability

Not applicable.
